# Extracellular Vesicles in the Progression and Therapeutic Resistance of Nasopharyngeal Carcinoma

**DOI:** 10.3390/cancers14092289

**Published:** 2022-05-04

**Authors:** Yunhan Shan, Peijun Zhou, Qin Zhou, Lifang Yang

**Affiliations:** 1 Department of Oncology, Key Laboratory of Carcinogenesis and Cancer Invasion of Ministry of Education, National Clinical Research Center for Geriatric Disorders, Xiangya Hospital, Central South University, Changsha 410078, China; 8211180819@csu.edu.cn (Y.S.); 196511043@csu.edu.cn (P.Z.); zhouqin5796@csu.edu.cn (Q.Z.); 2 Cancer Research Institute, School of Basic Medicine Science, Central South University, Changsha 410078, China; 3 Xiangya School of Medicine, Central South University, Changsha 410013, China

**Keywords:** extracellular vesicles, tumor microenvironment, therapeutic resistance, nasopharyngeal carcinoma

## Abstract

**Simple Summary:**

The occurrence and development of nasopharyngeal carcinoma (NPC) is closely related to the Epstein–Barr virus. Extracellular vesicles (EVs) play a critical role in tumor progression. EVs in NPC, especially EV-loaded molecules with Epstein–Barr virus characteristics, can not only allow the evaluation of the malignant degree and progression of a tumor but also provide appropriate methods and monitoring means for the therapy. The present review summarizes the pivotal role of tumor-derived EVs in regulating NPC progression and therapeutic resistance. Furthermore, the bench-to-bedside translations of EVs as biomarkers in the diagnosis and precise treatments of NPC are discussed.

**Abstract:**

Nasopharyngeal carcinoma (NPC) is an epithelial malignancy largely associated with Epstein–Barr virus (EBV) infection, which is frequently reported in east and southeast Asia. Extracellular vesicles (EVs) originate from the endosome or plasma membrane, which plays a critical role in tumor pathogenesis for their character of cell-cell communication and its cargos, including proteins, RNA, and other molecules that can target recipient cells and affect their progression. To date, numerous studies have indicated that EVs have crucial significance in the progression, metastasis, and therapeutic resistance of NPC. In this review, we not only summarize the interaction of NPC cells and the tumor microenvironment (TME) through EVs, but also explain the role of EVs in radiation and drug resistance of NPC, which poses a severe threat to cancer therapy. Therefore, EVs may show great potential as biomarkers in the early diagnosis of interfered targets of NPC therapy.

## 1. Introduction

Nasopharyngeal carcinoma (NPC) is an epithelial malignancy that arises from the nasopharyngeal mucosal lining. A report from the International Agency for Research on Cancer revealed that up to 133,354 new cases and 80,008 new deaths of NPC occurred in 2020, and more than 70% of new cases were reported in east and southeast Asia, which presented a sharp imbalance in geographical distribution worldwide [1]. Studies have shown some crucial risk factors that may contribute to the development of NPC, including Epstein–Barr virus (EBV) infection, host genetics, and environmental factors [2]. Several genetic susceptibility genes were found to promote NPC risk. For example, HLA genes located at the MHC complex on chromosome 6p21 were demonstrated as major risk loci inducing NPC oncogenesis [3]. As one of the most well-known pathogens, EBV could destroy human B-cell development to maintain a continuous infection [4].

Apparently, EBV was identified to regulate cell proliferation and has a close connection with the development of multiple malignancies and primarily of lymphoid and epithelial cell origin, such as NPC, parotid gland carcinoma, Hodgkin’s lymphoma, Burkitt’s lymphoma, post-transplant lymphoma, and a proportion of gastric carcinoma [5]. During the latent period of infection in NPC, EBV releases various products, including Epstein–Barr nuclear antigen 1 (EBNA1), latent membrane protein 1 (LMP1), LMP2, EBV-encoded small RNAs (EBERs), and miRNA (EBV-miR-BART). Specifically, LMP1 was recognized as the principal oncogene due to its transforming capacity in cell lines and the high expression in EBV-associated cancers [6].

Radiation therapy (RT) is the preferred and a powerful treatment for NPC [2]. Ionizing radiation (IR), considered the first and standard treatment in plenty of malignant tumors, usually induces cell death through unrepaired DNA damage to achieve its anti-tumor effects. Despite the high sensitivity of gross tumor tissue, radio-resistance still frequently occurs in NPC, thus resulting in NPC local recurrence and distal metastasis [7]. In most conditions, early-stage NPC is merely treated with radiotherapy. However, loco-regionally advanced tumors demand combined therapies such as chemo- or immune-therapy rather than single irradiation. Clinical trials have confirmed the tremendous benefits of concurrent chemoradiotherapy or adjuvant chemotherapy before or after radiotherapy versus radiotherapy alone in locoregionally-advanced NPC survival [2]. However, the resistance of NPC to chemoradiotherapy is still a clinical problem [8].

The tumor microenvironment (TME) consists of cancer cells, stromal cells, immune cells, and other complex components. TME can alter the intercellular communication between NPC cells and surrounding stromal cells and promote the cell proliferation, invasion, and metastasis of tumor cells [2]. Tumor or stromal cells may secrete soluble factors targeting TME components, while the TME allows a small number of tumor cells to evade apoptosis reversely, which consequently induces drug resistance [9]. In general, environment-mediated therapeutic resistance happens temporarily during the contact of tumor cells with the TME, and therapeutic sensitivity reverts after removing the cancer cells from the TME [10].

Extracellular vesicles (EVs) biologically function in cell-to-cell communication. Researchers have extracted and analyzed the different cargos of EVs to reveal their underlying mechanism and provide insight into the therapeutic application of environment-mediated therapeutic resistance in NPC [11]. It is believed that EVs will show excellent efficiency in clinical utility with the increasing evidence. In this review, the pivotal role of tumor-derived EVs (TDE) in regulating NPC progression and therapeutic resistance was summarized. Furthermore, we focus on the comprehensive functions of TME-derived EVs in NPC and discuss the bench-to-bedside translations of exosomes as biomarkers in the diagnosis and precise treatments of NPC.

## 2. Biogenesis and Secretion of EVs in NPC

Many cell types can release various EV types, and this process has been maintained throughout the evolution from bacteria to humans [12]. In terms of their biochemical composition, EVs are surrounded by a phospholipid membrane that contains significant levels of cholesterol, sphingomyelin, and ceramide and contains a decontamination-resistant membrane structure domain [13]. Traditionally, EVs are roughly classified into three categories based on differences in biogenesis: exosomes, microvesicles, and apoptotic vesicles [14]. However, EV standard classification is still controversial. The current limitations of classification include isolation, size, density, morphology, and lipid composition [13]. Exosomes are derived from the endosomal system. They are formed by inward budding of the limiting membrane of multivesicular bodies (MVBs), resulting in the generation of intraluminal vesicles (ILVs). MVBs are late endosomes containing large amounts of ILVs that fuse with the cell membrane, thereby releasing the ILVs as exosomes outside the cell. Alternatively, the MVBs fuse with the lysosome if the content is destined for degradation [15]. However, microvesicles originate from outward budding at the plasma membrane. Various molecular rearrangements in the plasma membrane support the biogenesis of microvesicles, including changes in lipid composition and protein components, and are also related to the metabolic capacity and Rho GTPase signaling pathway [16].

The formation of ILVs is the initiation of the biogenesis of exosomes. There are two important steps in the formation. Firstly, the endosome membrane is reconstituted into highly enriched tetraspanins; it is believed that CD63 and CD9 are the two tetraspanins playing a critical role in exosome formation [17]. Secondly, endosomal sorting complexes (ESCRTs) required for transport are recruited at the sites of ILV formation. ESCRTs are complexes composed of four independent proteins, ESCRT 0, I, II, and III. In the initial stages, ubiquitinated proteins in late endosomes are recognized and retained by Escrt-0. Subsequently, ESCRT-I and ESCRT-II complexes are recruited to the cytosolic side via stimuli. ESCRT-III drives vesicle scission, and the accessory proteins (such as VPS4) allow dissociation and recycling of the ESCRT machinery [18]. The EBV-LMP1 plays an important role in NPC progression and is also involved in intercellular interactions. The study found that the knockdown of ESCRT-III impaired the packaging and secretion of LMP1 into EVs [19]. CD63 is necessary for effectively packaging LMP1 into EVs; the knockout of CD63 led to the decrease in packaging and secretion of LMP1 [20].

Another participant ILV formation pathway is the syndecan-syntenin-ALIX pathway [21]. In this pathway, the interaction of syntenin with ALIX promotes ILV formation, a process that also depends on the availability of ALIX and ESCRTs [22,23]. Dingani Nkosi demonstrated that, after the knockout of Syntenin-1, Alix, HRs, and TSG101, the level of packaging and vesicle formation of LMP1 EVs decreased, and the decreased expression of Syntenin-1 and HRS was accompanied by changes in internal lysosome transport [19]. The results may clarify how LMP1 utilizes the host syndecan-syntenin-ALIX pathway machinery to direct the secretion of viral oncoproteins into the EV pathway.

The EBV can evade proteomic and lysosomal degradation pathways through secreted LMP1 [20]. Meanwhile, LMP1 also participates in secretion and loading by regulating CD63 and other molecules in NPC cells [24]. LMP1 protein has six transmembrane domains, which participate in transport and self-aggregation by recruiting tumor necrosis factor (TNF) receptor-related factors (TRAFs) in the cytoplasmic C-terminal activation region (CTARs) [25]. The internalization of LMP1 into MVB requires dissociation from TRAF2, which dissociates from LMP1 on the endoplasmic membrane and does not enter the exosome [26]. Nkosi et al. found that deletion mutants containing only N-terminal and transmembrane fragments 1 or 1 and 2 (TM1ΔC or TM1-2ΔC) were effectively transmitted to EVs and showed a similar intranuclear localization to the intact LMP1. The TM1 of LMP1 has multiple leucine residues that act as endosomal sorting motifs, targeting LMP1 in the endosomal pathway and ultimately translocating to EVs [27]. The study also found that LMP1 promoted the binding of ESCRT-0 and syntenin-1 and the EV secretion by affecting protein expression, such as ESCRT and syntenin [19]. Our team found that LMP1 upregulates syndcan-2 (SDC2) through the NF-κ B signal to promote EV formation in NPC cells, and the interaction of SDC2 with syntenin promoted the biogenesis of EVs [28]. LMP1 regulates the mTOR signaling pathway by interacting with mTOR, NEDD4L, and PP2A and then dephosphorylates ULK1 kinase, which is upstream of autophagy, thereby inhibiting autophagy, which is conducive to the formation of EVs [24]. Studies have shown that CD63-dependent vesicle protein secretion can resist the activation of mTOR and other signals downstream of LMP1. Destroying the normal autolysosome process will inhibit the signal transduction of viral protein and increase the secretion of LMP1. For example, after the knockout of CD63, mTOR activation is closely related to the formation of serum-dependent autophagy vacuoles, indicating that autophagy plays an important role in the secretion and regulation of exosomal LMP1 [29].

Another EBV encoded protein, latent membrane protein 2A (LMP2A), is related to the viral latency and pathogenesis of infected cells. Cholesterol consumption leads to the accumulation of LMP2A in the plasma membrane, which promotes the increase of LMP2A in exosome secretion [30]. Of note, EBERs and EBV-miR-BART are the most abundant untranslated viral RNAs and miRNA in latently EBV-infected cells and can be transferred via exosomes; thus, suggesting a microvesicular viral small RNA and microRNA transfer. These small RNAs and microRNA could confer resistance to apoptosis, modulating the innate immune response, angiogenesis, and other biological functions of receptor cells [31,32,33,34].

EV release following fusion of the membrane and plasma membrane of MVB is highly selective, and the sorting pathway is key to distinguishing exosomes from plasma membrane-derived EVs [35]. Rabs proteins are important regulators of intracellular vesicle transport in cells; they participate in vesicle budding, cytoskeleton interactions, and membrane tethering of the receptor compartment. The Rabs family consists of over 60 GTPases, each of which is preferentially associated with one intracellular compartment. Silencing of Rab27A was found to reduce the secretion of EVs in various tumor cell lines, including melanoma, breast cancer, head and neck cancer, and prostate cancer [36]. On the contrary, in the human breast cancer cell line MDA-MB-231, silencing of RAB27A did not show reduced exosome secretion, but the co-deletion of RAB27A and RAB27B did reduce the amount of secreted EVs [37]. The preliminary work of our group also found that the knockdown of Rab27A could significantly inhibit EV secretion in NPC [38]. Another work of our group showed that the exosomes secreted by LMP1-positive NPC cells were more than the exosomes secreted by LMP1-negative NPC cells. Mechanistically, LMP1 promotes exosome secretion through SYTL4 [28]. Indeed, SYTL4, an effector molecule of RAB27A, primarily promotes the fusion of vesicles with cell membranes to secrete EVs out of the extracellular environment [39].

**Figure 1:**
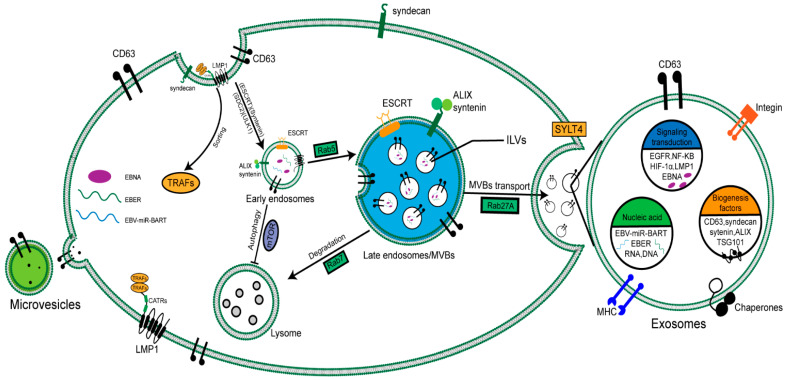
Biogenesis and secretion of extracellular vesicles in EBV-associated NP.

The above studies on the biogenesis and secretion of EVs in NPC fully illustrate the important role of virus-encoded proteins, RNA, and miRNA, especially LMP1, in regulating the formation and secretion of EVs in NPC cells (Figure 1). Meanwhile, it also shows that tumor- or virus-derived EV-loaded molecules participate in the communication between tumor cells and TME cells.

## 3. Roles of TDE in NPC

### 3.1. TDE Regulates NPC Cell Proliferation

TDE-derived proteins and miRNAs play a positive role in NPC growth. EVs isolated from NPC cells and packaged in PFKFB3 increased cell proliferation by regulating the cell cycle pathway and apoptosis through the activation of ERK and AKT pathways [40]. Exosomal miR-301a-3p enhanced the proliferative abilities and colony-formation efficiency of NPC cells by targeting BTG1 mRNA [41]. Our work showed that NPC cells containing LMP1 exhibited higher proliferation ability than cells without it. LMP1 increased the expression of SDC2 and SYTL4 through NF-κB signaling, which improves the formation and secretion of EVs. Increasing the quantity of EVs could facilitate the proliferation of recipient NPC cells. Subsequently, the animal experiment result showed that LMP1 improves the secretion of EVs and enhances tumor growth in vivo [28]. Taken together, these findings indicate the effects of TDEs on NPC cell proliferation.

### 3.2. TDE Promotes Tumor Metastasis

Metastasis is not only the main obstacle in the clinical treatment of NPC, but also the main cause of death in NPC patients. As a key pathological transformation procedure in cancer metastasis, epithelial-mesenchymal transition (EMT) decreases epithelial markers and intercellular junctions, resulting in a loss of epithelial polarity and reducing intercellular adhesion [42]. The researchers found that TDE PFKFB3 induced EMT in NPC cells, which increased NPC migration and invasion [40]. Li et al. demonstrated that EVs could transfer EGFR between highly metastatic NPC cells and poorly metastatic NPC. After that, EV-mediated EGFR overexpression downregulates ROS levels through the PI3K/Akt signal pathway, thereby promoting NPC cells with low metastatic potential to achieve an ability similar to NPC cells with high metastatic potential [43]. Nanbo et al. demonstrate that type III latency exosomes, when added to recipient-mediated cells, increased proliferation and upregulation of ICAM-1 more than EBV-negative exosomes and type I latency exosomes [44]. Studies have shown that EV and packaged cargo modified by LMP1 could change gene expression in recipient cells, thus affecting the phenotype of recipient cells and TME. N-cadherin and E-cadherin produced by LMP1 are related to EMT. Meanwhile, the expression of MMPs is associated with the degradation of extracellular matrix (ECM), thus participating in the remodeling of TME [45,46,47]. Shan et al. found that hypoxia increased the MMP13 levels in TDE in a HIF-1α-dependent manner and transferred MMP13 between normoxic and hypoxic cancer cells, thus remodeling the TME and inducing EMT migration and invasion of recipient NPC normoxic cells [48]. HIF1α localization in the exosomes does not depend on the viral oncogene. It was found that HIF1α was detected in the EVs of LMP1-negative cells. However, the presence of LMP1 significantly increases HIF1α in EVs [49].

## 4. Roles of TDE in the NPC Microenvironment

Here, we focus on the role of EVs on the TME. The blood capillaries, immune cells, normal fibroblasts (NFs), and ECM surrounding the cancer cells constitute the TME. As a medium, EVs improve the communication between tumor cells and the TME.

### 4.1. The Effects of TDE on Activating NFs of NPC Microenvironment

Cancer-associated fibroblasts (CAFs) are the primary stromal cells in the TME and are essential for cancer progression [50]. The results of our group showed that EV-loaded LMP1 secreted by NPC cells upregulated the expression of a-SMA and FAP by activating the NF-κB pathway in NFs. This process activates NFs into CAFs. Further studies showed that activated CAFs upregulate MCT4 through the NF-κB pathway and promote lactic acid and β-HB secretion, while MCT1 enhances tumor cell abilities to respire and fuel anabolism. The metabolic coupling of CAFs with tumor cells promotes the proliferation and IR resistance of NPC cells [38].

### 4.2. The Effects of TDE on Immune Cells

In recent years, the involvement of tumor-secreted EVs in TME immune regulation has been widely described. In breast cancer, EV-mediated immunosuppression regulates tumor proliferation, metastasis, and chemoradiotherapy resistance by affecting TME—especially in the formation of the pre-metastasis niche [51]. Here, we focus on the important roles of two kinds of immune cells (myeloid suppressor cells and T lymphocytes) related to TDE in NPC progression and immunosuppression.

Myeloid-derived suppressor cells (MDSCs) are related to the suppression of anti-tumor immunity. MDSCs are the precursors of myeloid-derived immune cells, including macrophages, dendritic cells, and neutrophils [52]. Monocyte-derived macrophages are among the most important immune cells since they have innate and acquired immune responses to pathogens and play a significant role in tissue homeostasis. Tumor-associated macrophages (TAMs) are the most abundant immune cell types in solid tumors. They can be affected by factors secreted by tumors, resulting in many inflammatory factors, such as IL-6, which can promote the occurrence and development of tumors [53]. For example, human hepatocellular carcinoma (HCC) cells can promote macrophages to secrete IL-6, thus inducing the expansion of HCC stem cells [54]. Moreover, colorectal cancer cells could recruit TAMs by secreting CCL20, producing abundant IL-6 [55]. In NPC, TDE has a stronger macrophage induction ability than exosomes derived from normal epithelial cells. It is found that only TDE can induce macrophages to produce IL-6, while exosomes derived from nasopharyngeal epithelial cells cannot; this difference may be due to the loading of different cargo [56]. Microglia is a type of glial cell that corresponds to macrophage in the brain and spinal cord. It’s the first and primary immune defense line of the central nervous system. NPC is a malignant cancer with high rate of metastasis which intrudes into the intracranial space by invading the skull base or via brain metastase. But it’s rarely observed brain parenchyma invasion and brain metastases of NPC patients [57]. miR-196a-5p take participant in the formation of the TME by directly inhibiting ROCK1 and further influencing microglial proliferation, phagocytosis and the release of inflammatory cytokines [58].

T-lymphocytes are derived from bone marrow multipotent stem cells [59]. It was found that TDE derived from NPC can affect the proliferation and differentiation of T cells by changing the phosphorylation levels of ERK and STAT in T cells. Furthermore, high exosomal protein levels were positively correlated with lymph node metastasis and shorter disease-free survival [60]. TDE contains tumor-specific antigens, tumor-related proteins, and immunosuppressive molecules, such as FasL, CD95L, TRAIL, and galectin-9, promoting T cell apoptosis [61,62]. Galectin-9 contacts TIM-3 receptors on the surface of exosomes and T cells to induce apoptosis. Interestingly, CD4 T cells specific for non-EBV antigens are sensitive to exosomes loaded with galectin-9 [63]. Since the galectin-9/TIM-3 pathway is involved in producing CD4 + CD25 + regulatory T cells, exosomal galectin-9 may contribute to T-cell proliferation in the tumor and peripheral blood of NPC patients [64]. At present, there is much evidence that in a variety of tumors, TDE can damage the function of immune cells (including T cells, NK cells, and dendritic cells) by enriching protein molecules such as galectin-9 and TGFβ and then inducing tumor immunosuppression [62,63,65,66]. Mrizak et al. found that TDE could recruit Treg cells into the TME of NPC by transporting chemokine CCL20. TDE also recruits conventional CD4 T cells and induces them to transform into suppressive Tregs. This study first reported the unique immunomodulatory ability of TDE to induce Treg amplification and upregulate its suppressive function [67]. Ye et al. demonstrated that exosomal miR-24-3p repressed FGF11 expression to mediate the phosphorylation of the ERK and STAT proteins, thereby inducing T cell dysfunction [68].

### 4.3. The Effects of TDE on Angiogenesis

Much evidence shows that TDE-mediated communication facilitates tumor cells to regulate the TME and increase tumor cell proliferation, invasion, and metastasis [69]. Meanwhile, endothelial cells are the main participants in angiogenesis and the communication target cells mediated by TDE. Previously, several studies had reported the function of TDE to promote angiogenesis in different cancers [70,71]. The NPC-derived exosomes loaded with PFKFB3 could promote angiogenesis [40]. Chan et al. found that NPC-derived exosomes could significantly induce the angiogenesis, migration and invasion of vascular endothelial cell HUVECs [72]. Exosomal MMP13 from NPC could mediate the TME by interaction with stromal fibroblast cells (HSF) and HUVECs, further facilitating the migration and invasion of NPC cells [73]. HMGB3-containing exosomes derived from NPC could be ingested by HUVECs, which could remarkably enhance tumor metastasis by inducing angiogenesis [74].

Moreover, non-coding RNAs also play a key role in angiogenesis, and microRNA is most commonly studied. miR-144 carried by TDE of NPC could contribute to angiogenesis via inhibition of FBXW7 and promotion the HIF-1α-dependent vascular endothelial growth factor (VEGF-A) of HUVEC cells [75]. miR-17-5p derived from NPC could inhibit BAMBI in HUVEC cells, thereby enhancing the expression of Akt/VEGF-A and promoting angiogenesis in TME [76]. It is worth noting that TDE-mediated miR-9 delivery inhibited angiogenesis in NPC by reducing the secretion of MDK and regulating the PDK/Akt pathway in HUVECs [77]. Overexpression of miR-23a in NPC could be transported to endothelial cell HUVECs through EVs and promote angiogenesis by directly targeting TSGA10, further regulating tumor growth. TDE-miR-23a may be beneficial for the early diagnosing and future predicting of metastases [78]. A recent study has shown that NPC-derived EV-loaded EBERs could be transferred to endothelial cells, subsequently inducing angiogenesis by stimulating VCAM-1 [33].

## 5. Roles of Microenvironment-Derived EVs in NPC

TME-derived EVs play an important role in the development of NPC. As the most important component of TME, CAF plays a key role in building a microenvironment conducive to tumor initiation, diffusion, angiogenesis, and metastasis by producing a variety of ECM proteins and regulatory molecules. An improved understanding of CAF biology will contribute to a deeper insight into how CAF affects the dynamic complexity and functional ductility of TME [79]. The density of CAFs was significantly correlated with the T stage in NPC and the significantly longer mean overall survival for patients with a low density of CAFs than patients with a high density of CAFs. Further, the density of CAFs in metastatic tissue of NPC was also significantly higher than that in the primary area. Therefore, CAFs in mesenchymal play an important role in the tumorigenesis, metastasis, and prognosis of NPC patients [80]. After the FGF19-loaded exosomes secreted by MSCs were ingested by recipient NPC cells, they could promote EMT through the FGFR4-dependent ERK signaling pathway to stimulate the progress of NPC [81].

miR-18a from M2 macrophages stimulates the progression of NPC cells by inhibiting TGFBR3. Further, upregulated miR-18a in NPC tissues is closely related to tumor lymph node metastasis and tumor size [82]. In fact, studies on a variety of malignancies, including ovarian cancer, colitis-associated colorectal cancer, and hepatocellular carcinoma, have found that knocking down miR-18a inhibits the progression of tumor cells [83,84]. In addition, the study also found that exosomes secreted by MSC overexpressing miR-34c significantly inhibit the proliferation of NPC cells, while the knockdown of miR-34c significantly improves the viability of NPC cells [85].

**Figure 2:**
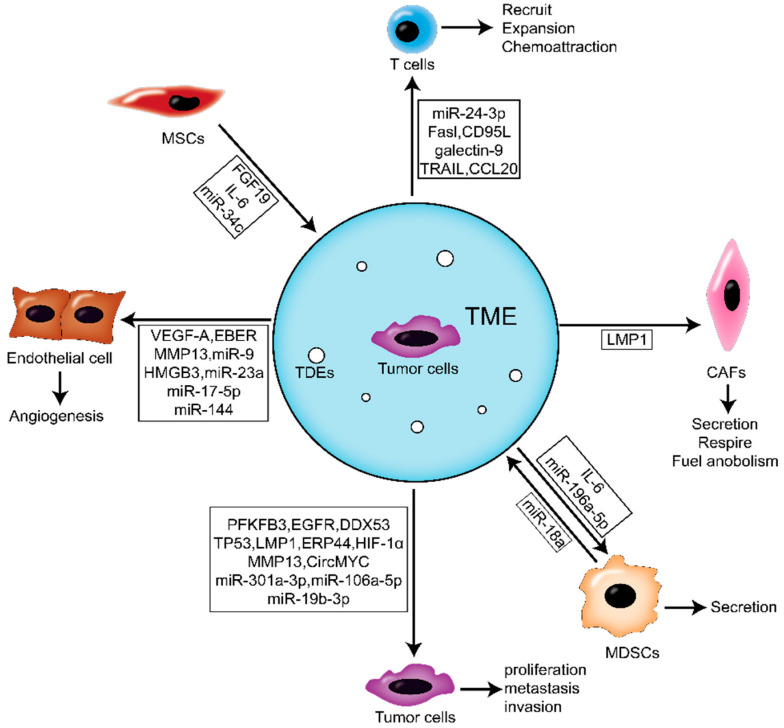
A schematic representation of the regulation of the EVs content from different sources on NPC cells and TME cells.

The above studies fully show that the TDE of NPC or EVs derived from cells of TME are involved in tumor proliferation and metastasis through various sources of transport, including various active molecules of cells or viruses (Figure 2). This has provided many meaningful molecular targets and interventions for the clinical diagnosis and treatment of NPC.

## 6. The Role of EVs in Chemoradiotherapy Resistance of NPC

Radiotherapy is the mainstay treatment modality for NPC, which is highly sensitive to IR. However, the main obstacle to radiotherapy is the inherent and acquired radiation resistance of cancer cells. miRNA-19b-3p upregulates the radiosensitivity of NPC by targeting the TNFAIP3/NF-κB axis [86]. Recently, TDE inhibit HGF/c-Met and EGF/EGFR pathways through miRNA-142-5p transmission, accelerating the radiosensitivity of NPC cells [87]. IR causes DNA double-strand breaks (DSB) and leads to fatal injury. Furthermore, in most cell types, IR-induced DSB triggers DNA damage with NF-κB activation as an antiapoptotic factor. Thus, inhibiting NF-κB activation could increase radiosensitivity [88]. p53-regulated apoptosis is considered the main cause of cell death induced by IR. p53 will activate the DNA repair system to maintain the integrity of the entire genome when DNA damage is caused by IR or cytotoxic drugs. If DNA damage is irreparable, it will initiate apoptosis [89,90]. In NPC, circMYC can promote cell proliferation and reduce radiosensitivity. Exosomal circMYC is a biomarker to distinguish radioresistant patients from radiosensitive NPC patients [91]. Our group reported that EVs derived from LMP1-positive NPC cells could mediate the radiation resistance and inhibit apoptosis in recipient cancer cells through the activated P38 MAPK pathway. Inhibition of P38 activity restored radiosensitivity in LMP1-positive EV-transformed NPC cells [92]. On the contrary, miR-34c promoted apoptosis of NPC cells by targeting β-catenin and inhibited resistance to radiotherapy. Therefore, inhibiting β-catenin is an effective means to inhibit tumor progression and restore radiosensitivity [85].

Chemotherapy has proven to be an effective treatment for NPC combined with radiotherapy [93,94,95]. Drug resistance is one of the most destructive problems in NPC treatment [96]. Platinum-based concurrent chemoradiotherapy is the standard treatment for patients with locoregionally advanced NPC. In addition, gemcitabine and cisplatin also have specific clinical effects [97,98]. circMYC has a strong association with the response to drugs [99]. It is reported that the overexpression of circMYC promoted the proliferation of human melanoma cells. Similarly, circMYC is closely related to tumor size, lymph node metastasis, and the TNM stage of NPC and can be used as an independent predictor of survival and disease recurrence in NPC patients [100]. Docetaxel has strong anti-tumor activity in NPC, and the combination of docetaxel and cisplatin has a high anti-tumor effect in metastatic NPC [101]. Yuan et al. found that compared to NPC cells, DDX53 was highly expressed in taxol-resistant NPC cells. Besides, the IC50 to taxol was significantly increased after treating the taxol-resistant cell line CNE1-TR exosomes compared with CNE1 cells. Exosome-mediated DDX53 from CNE1-TR cells was transferred into CNE1 cells and might promote resistance to taxol for NPC by upregulating with MDR1 [102]. Xia et al. found that overexpression of ERp44 could reduce cisplatin sensitivity by inhibiting apoptosis and pyroptosis. More importantly, under ERS, NPC cells produce exosomes containing ERp44, which can be transferred to adjacent cells to enhance chemical resistance [103]. Similarly, Li et al. found the transfer of miR-106a-5p by exosomes from cisplatin-resistant cells to susceptible NPC cells and conferred cisplatin resistance to recipient NPC cells. Mechanistically, miR-106a-5p targets the inhibition of ARNT2 expression, promotes NPC cell proliferation, migration, and invasion, and gives resistance to cisplatin treatment [104]. The exosomes secreted by drug-resistant endothelial cells can promote the proliferation, migration, and chemical resistance, as well as promoting the EMT of NPC cells [105].

## 7. Application of EVs in Diagnosis and Therapeutic Strategies

Although the combination of radiotherapy and chemotherapy has made significant progress, metastasis and recurrence are still difficulties in treating NPC. Due to the lack of specific early symptoms and the special pathogenic site, most NPCs diagnosed clinically are in the late stage. The antibody of EBV capsid antigen immunoglobulin A (VCA-IgA) is a known biomarker of NPC, but its positive rate is less than 70% [106]. At present, the most widely used TNM staging and EBV-DNA perform poorly in predicting distant metastasis of NPC, indicating the importance and urgency of exploring novel and more effective biomarkers. Due to the convenience of blood sampling, liquid biopsy based on protein characteristics may be a promising application field to explore NPC biomarkers. Exosomal miR-24-3p mediates T cell suppression by inhibiting FGF11 and participates in tumor pathogenesis. Therefore, exosomal miR-24-3p and its target gene FGF11 may be prognostic markers of NPC patients [68]. Jiang et al. revealed that circulating EV-derived miRNA profiles in NPC patients differed from healthy controls; mir-134-5p, miR-205-5p, miR-486-5p, miR-486-3p, and miR-409-3p were significantly dysregulated in NPC. Further, the diagnostic model composed of miR-134-5p, miR-205-5p, and miR-409-3p showed promising results in the diagnosis and prognosis of NPC [107]. CYPA plays an important role in many diseases such as kidney disease, cardiovascular disease, virus infection, and cancer by binding to membrane receptors and activating related signal pathways [108,109]. CYPA is a secretory protein, and Liu et al. revealed that the level of exosomal CYPA combined with EBV-VCA-IgA can be used to diagnose EBV-related NPC. It should be noted that CYPA as an independent indicator of NPC screening may not be ideal, which is probably due to the multiple functions of CYPA in normal physiological processes. Clinically, the combination of exosomal CYPA and EBV-VCA-IgA will improve the accuracy of diagnosis [110]. The exosomes loaded with viral BART miRNA secreted by NPC cells have sufficient stability and can spread from the tumor site to peripheral blood, which provides a basis for exploring it as novel tumor biomarkers [111]. In addition, Ramayanti et al. found that EV-loaded BART miR-13-3p in circulation is a meaningful, NPC-selective biomarker that can be used as part of the detection strategy of screening NPC in epidemic areas [112].

Many studies have shown the role of EVs in regulating TME, including changing the immune response and promoting tumor progression [113]. Further, studies have demonstrated that the increase of circulating EVs in the serum of patients with advanced cancer is related to disease progression to some extent [114,115]. NF-κB activated by LMP1 induces the growth, EMT, and metastasis of NPC by inhibiting miR-203 [116]. Studies have shown that miR-203 plays an important role in inhibiting several key steps of NPC, such as growth, EMT, invasion, and metastasis [117]. These effects can be reversed by using chemical inhibitors of NF-κB, such as aspirin. At present, aspirin has been used in the prevention and treatment of colorectal cancer [118]. Aspirin can significantly inhibit the secretion of LMP1 by NPC cells and promote the expression level of miR-203 in cells and EVs. The low expression of miR-203 in NPC was related to tumor stemness, chemoradiotherapy resistance, and poor prognosis in patients. This result indicates the potential novel use of low-cost and beyond therapeutic resistance aspirin in NPC [119].

The research shows that EVs can be divided into different subtypes according to the surface markers. Each subtype has unique function and clinical diagnostic significance. Using and developing new EVs detection technology for EVs subtype analysis is a meaningful work. Liu et al. established a method called PLA-RPA-TMA, which can detect EVs of LMP1+ or EGFR+ from NPC cells with high sensitivity and specificity, and found that they are effective markers for early diagnosis of NPC [120]. Different from normal cells, tumor cells expose phosphatidylserine (PS) at the outer leaflet of cell surfaces. The EVs subtype rich in PS+ was isolated by iodixanol density gradient centrifugation and considered as a tumor marker related to tumor growth and metastasis [121]. Recently, Domenico proposed an innovative phage display technology to identify, isolate and comprehensively analyze EVs based on the antigenic reactivity of different subtypes of EVs, this method will provide important technical support for personalized diagnosis and treatment of tumors [122].

With the in-depth study of various aspects of EVs related to NPC, the mechanism of tumor chemoradiotherapy resistance will be clarified from a new perspective and provide new intervention targets for targeted treatment resistance and new markers for the diagnosis of NPC (Table 1).

## 8. Conclusions

In summary, in the occurrence and development of NPC closely related to EBV, EVs promote the malignant proliferation, invasion, and therapeutic resistance of tumors through the communication with tumor cells or TME. EVs in NPC, especially EV-loaded molecules with EBV characteristics, can not only evaluate the malignant degree and progression of the tumor, but also provide appropriate methods and monitoring means for the therapy of NPC. However, there are still some issues to be further clarified. For example, the molecular mechanism of EVs in malignant progression, distant invasion, and therapeutic resistance of NPC needs to be explored. In addition, although considerable research evidence has effectively promoted the clinical application of EVs, there is still a lack of standardization regarding the subtype and extraction technology of EVs. Furthermore, there are still many technical problems to be solved, such as establishing fast, low-cost and high-purity EVs separation and identification methods to improve the sensitivity and specificity of detection technology. Meanwhile, which component of EVs is suitable for biomarkers and therapeutic targets? These also require a deeper understanding of the basic mechanisms and characteristics of EV biology in NPC and also need the support of clinical analyses and experimental data.

## Figures and Tables

**Table 1:** Summary of the EVs content and their role in NPC.

Function		Regulatory Mechanism	References
Radioresistance	miR-34c	Targeting β-catenin	[85]
	miR-19b-3p	Increasing NF-κB activity	[86]
	miR-142-5p	Inhibit HGF/c-Met and EGF/EGFR pathways	[87]
	p53	Activate the DNA repair system and promote apoptosis	[89]
	circMYC	Promote cell proliferation and reduce radiosensitivity.	[91]
	LMP1	Activate P38 MAPK pathway	[92]
Chemoresistance	circMYC	Promote cell proliferation and metastasis	[101]
	DDX53	Upregulate with MDR1	[102]
	ERp44	Inhibiting cell apoptosis and pyroptosis	[103]
	miR-106a-5p	Targeting ARNT2	[104]
Diagnosis and therapeutic	miR-24-3p	Mediating T-cell suppression via repression of FGF11	[68]
	CYPA	Marker of EBV-associated NPC	[110]
	BART13-3p	NPC-selective biomarker	[112]
	miR-134-5p, miR-205-5p, miR-486-5p, miR-486-3p, miR-409-3p	Significantly dysregulated in NPC	[107]
	miR-203	Targeting both CDH6 and RUNX2	[119]
